# User Satisfaction of Using Electronic Medical Record System and Its Associated Factors among Healthcare Professionals in Ethiopia: A Cross-Sectional Study

**DOI:** 10.1155/2023/4148211

**Published:** 2023-04-17

**Authors:** Abiy Tasew Dubale, Nebyu Demeke Mengestie, Binyam Tilahun, Agmasie Damtew Walle

**Affiliations:** ^1^Department of Health Informatics, College of Health Science, Mattu University, Metu, Ethiopia; ^2^Department of Health Informatics, Institute of Public Health, College of Medicine and Health Sciences, University of Gondar, Gondar, Ethiopia

## Abstract

**Background:**

Electronic medical record (EMR) systems have become essential for the proper management of patients' information. Electronic medical record systems are on the rise in developing countries due to the need to ensure improved quality of healthcare. However, EMR systems can be ignored, if users are not satisfied with the implemented system. User dissatisfaction has been associated with the failure of EMR systems as a primary factor. There is also limited research done in the Ethiopian context on EMR user satisfaction at private hospitals. This study is aimed at assessing user satisfaction with electronic medical records and associated factors among health professionals working at private hospitals in Addis Ababa.

**Methods:**

Institution-based cross-sectional quantitative study was conducted among health professionals working at private hospitals in Addis Ababa, from March to April 2021. A self-administered questionnaire was used to collect the data. EpiData version 4.6 and Stata version 25 were used for data entry and analysis, respectively. Descriptive analyses were computed for the study variables. Bivariable and multivariable logistic regression analyses were carried out to assess the significance of independent variables on dependent variables.

**Results:**

A total of 403 (95.33% response rate) participants completed all the questionnaires. More than half of 214 (53.10%) of the participants were satisfied with the EMR system. Factors associated with user satisfaction with electronic medical records were good computer literacy (AOR = 2.92, 95% CI: [1.16-7.37]), perceived information quality (AOR = 3.54, 95% CI: [1.55-8.11]), perceived service quality (AOR = 3.15, 95% CI: [1.58-6.28]), perceived system quality (AOR = 3.05, 95% CI: [1.32-7.05]), EMR training (AOR = 4.00, 95% CI: [1.76-9.03]), computer access (AOR = 3.17, 95% CI: [1.19-8.46]), and HMIS training (AOR = 2.05, 95% CI: [1.22-6.71]).

**Conclusions:**

In this study, health professionals' EMR satisfaction was moderate. The result showed that EMR training, computer literacy, computer access, perceived system quality, information quality, service quality, and HMIS training were associated with user satisfaction. Improving computer-related training, system quality, information quality, and service quality is an important intervention to improve the healthcare professional's satisfaction towards using electronic health record systems in Ethiopia.

## 1. Background

Globally, there is a rising demand for an efficient and trustworthy healthcare delivery system, such as eHealth and its subcategory electronic medical records (EMRs) system, which offers an infinite potential to cut back costs, advance health information exchange, and improve healthcare access [1]. Electronic medical records (EMRs) were computerized medical information systems to capture, store, process, and display patient information in the application of information and communication technology (ICT) [2, 3]. The EMR systems are used for the proper care of patient information (computer-based systems for entry, storage, presentation, retrieval, and printing of information contained in a medical record) [4]. This electronic medical system technology has many benefits, such as legibility and completeness of medical information and documentation, immediate access to information anywhere at any time, a large clinical database, and decision-support techniques with some limitations [5]. A study conducted in China revealed that satisfaction with EMR was 70.7% [6]. But from the study in the Saudi government hospital, physicians indicated that 40% were satisfied with the EMR system [5].

The adoption of electronic medical record (EMR) systems is on the rise in developing countries due to the need to ensure improved quality of healthcare systems [7]. But there were so many problems hindering the use of the system [8]. User satisfaction is one of the determinants for the expected future usage of EMRs. User satisfaction is characterized as a subjective response to the person's interaction with the EMR system and can be measured in different ways [9]. User satisfaction is the most significant predictor of long-term EMR performance and acceptance [10]. Many private hospitals are currently implementing EMR systems in Ethiopia, which is a critical time for the establishment of standards that can measure and allow user satisfaction with EMR systems.

The implementation of the electronic medical record system in health systems faces the challenge of substituting paper-based medical record systems [11, 12]. The implemented EMR system also has many problems hindering its use of the EMR system [8]. Using an implemented system has various factors; one of the factors was users' response or user satisfaction with the implemented EMR system [8].

Ethiopia's national eHealth policy attempts to direct and develop healthcare information and communication technology (ICT) solutions to reduce poor health information system issues in the healthcare sector [13, 14]. The Ministry of Health (MOH) has a vision of equitable, quality, and timely health services with eHealth and has recognized and identified eHealth as a key enabler of transformation, including EMRs [15].

Despite the commitment of healthcare systems to the implementation of EMRs, the assessment from a user's perspective is still lacking [16]. The health professional understanding of EMR is a key factor in the successful use of the EMR system [17]. Even though electronic medical records can improve users' work efficiency, the differences in experience, understanding, and skills can lead to dissatisfaction among health professionals and an inability to realize their full potential to use with EMRs [18]. User dissatisfaction with the EMR system has been associated with the failure of many systems. The implementation of new EMR systems can be also opposed if users are not satisfied with the system [19]. Electronic medical record user satisfaction varies from a developed country to a developing country. A study conducted in Ethiopian public hospitals indicates that EMR user satisfaction was 35.6% [20].

In Ethiopia, the current health sector transformation plan strengthens the health management information system and incorporates a computerized health management information system priority policy plans to ensure health service quality and equity [20]. Private health facilities have a key role to play in the health information revolution as well as the Ethiopian healthcare system. To increase the quality of health information, EMR adoption in the healthcare system is a primary enabler. Nowadays, in Ethiopia, most private hospitals implement EMR systems, especially in Addis Ababa. Studies on EMR system implementation settings are necessary to understand the critical success and failure factors. The determinant factors that affect EMR user satisfaction in private hospitals might be different from the factors in public hospitals. Thus, determining the level of user satisfaction and factors that affect user satisfaction is important for the sustainability of an implemented EMR system.

Hospitals play a significant role in the healthcare sector [21]. However, the management and organizational literature has investigated the differences between private and public organizations, for example, in terms of care quality, patient satisfaction, organizational climate, performance, and costs [22, 23]. A study done in the USA showed that private hospitals have a higher prevalence of adoption of EMR utilization than public hospitals [24]. Another study done in Greece revealed that high healthcare technology was largely emphasized in private hospitals and diagnostic facilities as a result of the private sector's explosive growth, which followed and responded to technological developments quickly, and it is significant that over 90% of private health investments are focused on advanced biomedical technology, compared to an estimated 30% of public health investments [25]. Moreover, a study done in Bangladesh revealed that the private hospital has a dedicated IT department, with qualified people, and ICT infrastructure and has already created, deployed, and utilized eHealth solutions [21]. Similarly, in Ethiopia, the adhocracy values like learning, improvement, experimentation, and the independence for employees regarding healthcare technologies to make their professional judgments are further strengthened by private healthcare administration than public hospitals. Hence, assessing the satisfaction level at private hospital is better. Although private hospitals of healthcare professionals used the EMRs, the satisfaction level of using the system in these hospitals is uncertain.

Different research tries to identify factors that contribute to user satisfaction; among those, users' ages and user background (experience, computer literacy, and training), IT qualification or IT course, perceived system quality, perceived information quality, perceived service quality, and perceived usability of EMRs are the determinant factors [7, 9, 26–28]. However, as per our knowledge, research on the level of user satisfaction with EMRs in Ethiopian private hospitals is not conducted. Therefore, this study is aimed at assessing the level of user satisfaction among health professionals working at private hospitals and identifying the factors affecting user satisfaction with EMRs. It will be a baseline for future investigation, the policymaker, and health program planner to use ensure health information quality.

## 2. Methods

### 2.1. Study Design and Period

An institution-based cross-sectional quantitative study design was conducted from March to April 2021 at the private hospitals in Addis Ababa, Ethiopia.

### 2.2. Study Area

The study was conducted in Addis Ababa, the capital city of Ethiopia. The health institutions in the city have 134 private primary clinics, 437 private medium clinics, 265 private specialty clinics, 117 government health centers, 12 public hospitals, and 28 private hospitals [29].

### 2.3. The Population of the Study and Sample Size Determination

All health professionals who are working at private hospitals in Addis Ababa were considered as the source population, whereas all health professionals who are working at private hospitals that implemented EMRs in Addis Ababa during the study period were considered as the study population. Health professionals who were working at private hospitals and used the EMR system in Addis Ababa were included in the study, but healthcare professionals who were not available during the study, had less than 6 months of work experience, and worked at the COVID-19 center during data collection time were not included in the study.

The sample size was determined based on the assumption of a single population proportion formula. Since the magnitude of user satisfaction in private hospitals is unknown, 50% prevalence, a precision of 5% and a 95% confidence interval, and a nonresponse rate of 10% were taken to calculate the sample size. Finally, the 423 sample size was estimated.

(1) $$\begin{array}{c} \frac{n=\left(z_{2}^{\alpha }\right)2\times p\left(1-p\right)}{d2},n=\frac{1.95^{2}\times 0.5\left(1-0.5\right)}{\left(0.05^{2}\right)}=384.2\\ n=384+\left(384.2\times 0.1\right)=423 \end{array}$$

where *n* is the estimated sample size, *p* is the proportion of user satisfaction, *z*_2_^*α*^ is the value (*Z*-statistic) at the 95% confidence level (*α* = 0.05) which is 1.96, and *d* is the margin of error 5% (0.05).

### 2.4. Sampling Technique and Procedures

Study participants were selected from private hospitals that use the EMR system in Addis Ababa, Ethiopia. 28 hospitals were obtained based on those currently licensed under the Addis Ababa Food, Medicine and Healthcare Administration and Control Authority (AA FMHACA) and from the Addis Ababa Health Bureau. For each private hospital, a proportional allocation of the participants was done. The participants were selected using a simple random sampling method from each hospital.

### 2.5. Measurement

#### 2.5.1. Health Professional

In this study, healthcare professionals included physicians, nurses, medical laboratory technicians and pharmacists, radiologists, and HMIS (health data entry and management secretaries and information system officers) [20, 30].

#### 2.5.2. Satisfaction

User satisfaction is users' level of overall satisfaction with their interaction with the EMR system. Satisfaction was assessed using a 5-point Likert scale with 5 questions. Items for the composite variables were scored on a Likert-type scale with a maximum score of 5 and a range of 1 for “strongly disagree” to 5 for “strongly agree.” Finally, the composite variable score was dichotomized as “yes” or “no.” Accordingly, the final scores of participants with a score equal to the median and above were categorized as yes; those scores below the median were categorized as no [20, 27].

#### 2.5.3. Perceived System Quality

System quality is a system's overall performance, as perceived by users. System quality was assessed using a 5-point Likert scale with a 6-item questionnaire, and participants were asked system quality questions; those who score median and above were perceived as having good system quality; those scores below the median were categorized as not having good system quality [20, 27].

#### 2.5.4. Perceived Information Quality

Information quality is the desirable characteristic of the system output. Information quality was assessed using a 5-point Likert scale with a 7-item questionnaire, and participants who score median and above were categorized as having good information quality; those who scored below the median were categorized as not having good information quality [20, 27].

#### 2.5.5. Perceived Service Quality

Service quality refers to the quality of the support that system users receive from the department and support personnel. Service quality was assessed using a 5-point Likert scale with a 9-item questionnaire; participants who score median and above were perceived as having good service quality; those who scored below the median were categorized as not having good service quality [20, 27].

#### 2.5.6. e-Health Literacy

In this study, eHealth literacy is defined as the health professionals' ability to locate and use credible information from the Internet. The level of eHealth literacy was measured using eight Likert scale questions with five responses ranging from “strongly agree” to “strongly disagree,” and then, the level of eHealth literacy was determined based on an eHealth literacy score of 26 which was used as a cutoff point. After a relevant literature review, we labeled eHealth literacy score ≥ 26 as high eHealth literacy and eHealth literacy score < 26 as low eHealth literacy [31, 32].

### 2.6. Data Collection Tool and Procedures

Self-administered questionnaires adapted from different literature were used [3, 20, 28, 33, 34]. The questionnaire has been categorized into three categories. The first section has sociodemographic and user background having 14 items; the second category has user satisfaction, system quality, information quality, and service quality, and to measure user satisfaction, 5 items are used, whereas 9 items are used to assess service quality, 6 to assess system quality, and 7 items to assess information quality. The third category has 10 items to assess system usability and 8 items to assess eHealth literacy. The questionnaire has a brief explanation of the purpose of the research to the participants. Participants were asked to volunteer during the study term, and those who consent were given questionnaires. Five health information technicians (HIT) were used for data collection, and one health officer (HO) who has experience in data collection supervised the data collection process.

### 2.7. Data Collection and Quality Control

The training was given to data collectors and supervisors on the objective of the study, data collection procedures, data collecting tools, participant's approach, data confidentiality, and participants' right before the data collection date. The supervisors and investigator checked the completeness of the questionnaires. Before the actual data collection, pretesting of the questionnaires was conducted among health professionals (10% of the total sample size) at the Washington Medical Center before the study period. The completeness and consistency of the data were checked before data collection, and then, necessary modifications were done based on the pretest findings. The reliability was obtained by calculating the value of Cronbach's alpha values that were all above the threshold value of 0.7 (*α* = 0.83). The questionnaires were checked for missing values and discrepancies and completeness.

### 2.8. Data Processing and Analysis

Data from participants was edited and cleaned manually before being entered into the software. A data entry template was created based on study variables using EpiData v. 4.6, and manually edited data were entered into the software for further editing and analyzed it using Stata v.15. Descriptive frequencies, mean, and standard deviations were summarized to describe the study population with relevant variables. The binary logistic regression analysis technique was used to analyze the association between the independent variable to the dependent variable (satisfied with EMR). Variables with a *p* value less than 0.2 in the bivariable analysis were entered into multivariable analysis for controlling the possible effects of confounders. The variance inflation factor (VIF < 10) was used to determine the multicollinearity existence among independent variables, and there was no issue with it. In addition, the Hosmer-Lemeshow goodness-of-fit test using *p* value > 0.05 was performed, and the result obtained 0.74. Variables that were significant on the best of adjusted odds ratio (AOR), with 95% CI and *p* value < 0.05, were considered to be the determinant factors of user satisfaction.

### 2.9. Ethical Clearance

Ethical clearance was obtained from the institutional review board of the University of Gondar with reference number IPH/1502/2013. Supporting letters were obtained from the Addis Ababa Food, Medicine and Healthcare Administration and Control Authority (AA FMHACA) and the Addis Ababa health bureau. After getting the letter of support from each private hospital, written consent was obtained from each study participant. The data collection procedure was anonymous, and their privacy was being kept. Attention was given to the researcher to not personalize any of the responses of the participants during data presentation, analysis, and interpretation. To keep confidentiality assurance to the study participants, any information was provided by them.

## 3. Results

### 3.1. Sociodemographic Characteristics of Participants

A total of 423 healthcare professionals from 11 private hospitals were approached, and 403 of them completed all the questionnaires with a 95.33% response rate. Of the total 403 participants, 227 (56.33%) were female. The age of the participants ranged from 21-69 years with a mean age of 31.4 (SD ± 7.12) years. Almost half of 198 (49.13%) of the participants were nurse professionals. Most of the participants 215 (53.35%) had working experience of fewer than five years (Table 1).

### 3.2. User Background and Technology-Related Factors

From the total participant, the finding of this study revealed that those participants who had adequate computer access were 159 (39.45%). In terms of eHealth literacy, 319 (79.16%) had high eHealth literacy, and 187 (46.7%) of them have received training on HMIS implementation. Participants who had good computer literacy were 166 (41.19%). Of the participants, only 9 (2.23%) worked part time. Of the participants, almost half of the participants 200 (49.63%) do not have good perceived service quality. Among the participants, the majority, 319 (79.16%), had high eHealth literacy (Table 2).

### 3.3. User Satisfaction Level of EMRs in Ethiopia

**Figure 1:**
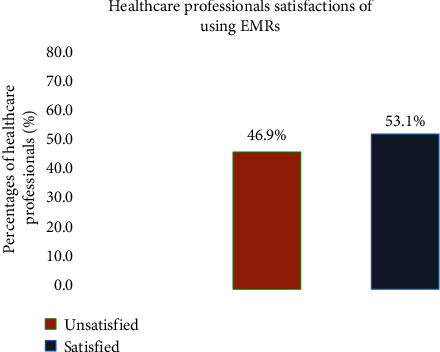
Proportion of user satisfaction towards using EMRs in Ethiopia, 2021.

Of the total participants, 214 (53.10%) (95.0%, CI: 48.10%-57.94%). were satisfied with the EMR system (Figure 1). The median score of satisfaction with using the electronic medical record system was 18 (IQR = 16–21), and the minimum and maximum scores were 5 and 25, respectively.

### 3.4. Factors Associated with User Satisfaction with Using EMRs in Ethiopia

The logistic regression analysis examined users' satisfaction with the electronic medical record systems and associated factors. After controlling the confounder, computer literacy, perceived system quality, perceived information quality, perceived service quality, computer access, HMIS training, and EMR training were significantly associated with user satisfaction towards EMRs (*p* ≤ 0.05).

Participants who reported having good computer literacy nearly 3 times (AOR 2.92, 95% CI: [1.16-7.37]) were more likely to be satisfied with EMR systems when compared with those who had reported poor computer literacy. The participants who had adequate computer access were 3.17 times (3.17, 95% CI: [1.19-8.46]) more likely to be satisfied with the EMR system than those who had inadequate computer access. Participants who received EMR training were 4 times (AOR 4.00, 95% CI: [1.76-9.03]) more likely to be satisfied with the EMR system than those not trained.

Participants who had received previous HMIS training were 2.86 times (AOR 2.86, 95% CI: [1.22-6.71]) more likely satisfied with the EMR system.

The participants who perceived the system to be of good quality were 3.05 times (AOR 3.05 95% CI: [1.32-7.05]) more likely to be satisfied with the EMR. Study participants who perceived the information to be of good quality were 3.26 times (AOR 3.26, 95% CI: [1.55-8.11]) more likely satisfied with EMR and perceived service to be of good quality were nearly 3 times (AOR 3.15, 95% CI: [1.58-6.28]) more likely satisfied (Table 3).

## 4. Discussion

This study is aimed at assessing user satisfaction with the EMRs and determinant factors, which affect the EMR system at private hospitals. The user satisfaction of healthcare professionals with electronic medical records (EMRs) is a helpful component for the quality of health information systems. To the best of our knowledge, no studies have been undertaken on user satisfaction of electronic medical records (EMRs) in Ethiopia's private hospitals. In this study, health professionals in private hospitals are frontline users of the EMR systems. The study revealed that the overall user satisfaction among health professionals working at private hospitals was 53.10% (95%, CI:48.10%-57.94%). This finding is lower than the study done in Ethiopia at Ayder Referral Hospital (66.9%) [8]. This disparity might be due to the number of health facilities or the research being done in a single center. Although the information technology infrastructure is different between private and public hospitals in Ethiopia, the Ayder public hospital is higher than in this study (private hospitals). The possible reason for this discrepancy might be due to the sample size used and study participants.

This result is also lower than studies done in China (70.7%) and Southern India (61.40%) [34, 35].

The possible explanation for this could be the infrastructural differences, the qualities of the EMRs, and the study setting. However, this study's finding is higher than the study done in Ethiopian public hospitals 35.6% [20]. This difference could be explained by the infrastructural difference, the most effective cultural orientation in terms of perceived individual performance improvement for private hospitals, the study period, and the quality EMR system at private hospitals. In addition, a higher study was done in Saudi governmental hospitals 40% [5]. The difference might be because of the study period and infrastructure. The other possible reason might be the difference in study participants and study setting.

Participants who had adequate computer access were more likely satisfied with EMR when compared to those with inadequate computer access. This showed that providers can now deliver more individualized care by customizing treatment programs for each patient because of computers and might be satisfied with the EMRs. The result is consistent with a study in Ethiopian public hospitals [8, 30], a study in Greek [26], and Saudi Arabia [36]. This could be explained by computer access which is a common issue in developing countries, but they know computers are having an increasingly significant impact on healthcare [20]. Accordingly, computer accessibility or adequate computer access may increase their motivation to use the EMR system and their satisfaction in Ethiopia.

In this study, the participants who had EMR training were more likely satisfied with the EMR system than those who had not trained. This study is consistent with a study in Saudi Arabia [36], a qualitative study in United Arab Emirates (UAE) [18], and Ethiopia [8, 20]. The possible reason for this could be that EMR training is more likely to increase participants' satisfaction with the EMR system [37]. Similarly, participants who had received previous HMIS Training were more likely to be satisfied with the EMR system. This showed that HMIS training could directly affect health professionals' performance to use the system and user satisfaction. Therefore, healthcare professionals, who are trained in the EMRs as well as HMIS, think that they will not encounter any issues using the system and consequently improve user satisfaction.

The participants who had good computer literacy were more likely satisfied compared to those who had poor computer literacy. This finding is in line with the studies in Saudi Arabia [37], United Arab Emirates (UAE) [18], and Ethiopia [20]. The level of use and satisfaction of healthcare providers towards the use of EMR depends on their computer literacy [37]. Similarly, a person with good computer literacy can easily and quickly adapt to the system than those who do not have computer literacy. As a result, most institutions require that healthcare professionals take a computer literacy course, hospitals offer ongoing training opportunities for staff members to improve their computer literacy, and healthcare personnel should be proficient in using EMRs and be satisfied with them as the industry moves more and more toward computerized systems to enable universal access to medical data and other outcomes.

The participants who perceived the system to be of good quality were more likely satisfied with the EMR system compared to those that are not good. This result is consistent with the study done in South Africa [38], Greece [26], and Ethiopia [20, 33]. This showed that higher system quality is expected to lead to higher user satisfaction and positive impacts on individual productivity [28]. System quality includes responsiveness, user interface, user-friendliness, and system usability, and the satisfaction levels of healthcare professionals toward using EMRs will therefore increase if these elements have a net beneficial impact [38].

The participants who perceived the information to be of good quality were more likely satisfied with the EMR system. The result of this finding is similar to a study conducted in Greece [26], Saudi Arabia [39], Nigeria [38], and Ethiopia [20, 33]. This implies that information quality like accuracy, completeness, currency, and format influence user perceptions about satisfaction [40]. Moreover, the quality of the information is essential, and if the information is not complete, up-to-date, clear, and secured, the quality of the data is reduced and end users are also not happy with the system. Therefore, managers should place special emphasis on the following elements when implementing EMR: making sufficient information available, ensuring good accuracy and timely updating of information on the system, and ensuring that reports are in a format and layout that health professionals regularly use and understand.

Participants who perceived service to be of good quality were more likely satisfied with the EMR system. This demonstrated that users' satisfaction and propensity to use the system will be higher when they perceive the good service quality of the EMRs. This result is in line with a study conducted in Turkey [41], Greece [42], Nigeria [38], America [43], and Ethiopia [20, 33]. The quality of the service can be improved by developing a welcoming environment that is attentive to user concerns. Moreover, computers are also to be placed within the hospitals so that physicians can enter patient data without having to wait for an available one. Improving service quality also involves providing immediate system support, and they must offer adequate assistance to increase the satisfaction level of healthcare professionals for using EMRs [33]. Therefore, EMR implementation managers need to focus considerably more on enhancing the service quality of EMR systems.

## 5. Conclusion

In this study, health professionals' EMR satisfaction was moderate. The result showed that EMR training, computer literacy, perception about system quality, information quality, service quality, and HMIS training influence user satisfaction. Those were considered important factors for improving the healthcare professionals' satisfaction towards using the electronic medical record system in Ethiopia. Moreover, increasing the health professionals' satisfaction with the EMR system is a prerequisite to increasing the health information revolution and quality of the healthcare system, so the government and other responsible bodies would be encouraged to use electronic medical record technologies in hospitals.

### 5.1. Limitations of This Study and Future Research

This study has some limitations; first, the study used a self-administered questionnaire, and most of the variables might have been exposed to social desirability bias or response bias. The second limitation is that it was not supported by qualitative findings, and the study was only in private hospitals which may affect the generalizability of the findings. Further study using an advanced model with six interrelated dimensions of the DeLone and McLean IS success model (D&M model) might be important to explore the associated factors of user satisfaction and net benefit.

## Figures and Tables

**Table 1:** Sociodemographic characteristics of health professionals working at private hospitals in Addis Ababa, Ethiopia, 2021.

Variable	Category	Number	Percent
Gender	Female	227	56.33
	Male	176	43.67
Age	21-30	226	56.08
	31-40	142	35.24
	>40	35	8.68
Profession	Physician	74	18.36
	Nurse	200	49.63
	Laboratory	49	12.16
	Pharmacy	39	9.68
	Radiology	15	3.72
	HMIS staff	26	6.45
Experience in year	≤5	215	53.35
	6-10	128	31.76
	>10	60	14.89
Salary (ETB)	<5,000	98	27.76
	5,000-10,000	56	15.86
	10,000-15,000	79	22.38
	>15,000	120	33.99

**Table 2:** Information technology (IT) qualification, EMR training, HMIS training, working part time, and technological factors among health professionals working at private hospitals in Addis Ababa, Ethiopia, 2021.

Variable	Category	Number	Percent
Computer access in the hospital	Adequate	277	68.73
	Inadequate	176	31.27
EMR training	Yes	215	53.35
	No	188	46.65
HMIS training	Yes	216	53.60
	No	187	46.40
IT qualification	IT qualified	332	82.38
	Not IT qualified	71	17.62
Working permanently	Yes	394	97.77
	No	9	2.23
Computer literacy	Poor	166	41.19
	Good	237	58.81
Perceived system quality	Not good	176	43.67
	Good	227	56.33
Perceived information quality	Not good	182	45.16
	Good	221	54.84
Perceived service quality	Not good	200	49.63
	Good	203	50.37
Perceived system usability	Low	220	54.59
	High	183	45.41
eHealth literacy	Low	84	20.84
	High	319	79.16

**Table 3:** Bivariable and multivariable analyses of factors associated with user satisfaction of electronic medical records among health professionals working at private hospitals in Addis Ababa, Ethiopia, 2021.

Character	EMR satisfaction, *n* (%)		COR (95% CI)	AOR (95% CI) *p* value	
	Satisfied	Dissatisfied			
Sex					
Male	91 (51.70)	85 (48.30)	1.00		
Female	123 (54.19)	104 (45.81)	1.10 (0.74-1.64)	1.17 (0.58-2.36)	0.64
Age					
21-30	122 (53.98)	104 (46.02)	2.05 (0.58-7.20)	0.55 (0.14-2.10)	0.38
31-40	78 (54.93)	64 (45.07)	2.13 (0.59-7.61)	1 .51 (0.13-2.02)	0.34
>40	14 (36.36)	21 (63.64)	1.00		
Computer literacy					
Good	191 (80.59)	46 (19.41)	25.80 (14.96-44.54)	2.92 (1.16-7.37)	0.023^∗^
Poor	23 (13.86)	143 (86.14)	1.00		
Perceived system quality					
Good	183 (82.81)	38 (20.11)	23.45 (13.93-39.49)	3.05 (1.32-7.05)	0.009^∗∗^
Not good	31 (17.19)	151 (82.97)	1.00		
Perceived information quality					
Good	187 (82.02)	41 (17.98)	25.00 (14.69-42.53)	3.54 (1.55-8.11)	0.003^∗∗^
Not good	27 (15.43)	148 (84.57)	1.00		
Perceived service quality					
Good	162 (79.80)	41 (20.20)	11.24 (7.06-17.92)	3.15 (1.58-6.28)	0.001^∗∗^
Not good	52 (26.00)	148 (74.0)			
Profession					
Physician	38 (17.76)	36 (19.05)	1.00		
Nurse	94 (43.93)	106 (56.08)	0.84 (0.49-1.43)	0 .62 (0.24-1.61)	0.332
Laboratory	34 (15.89)	15 (7.94)	2.14 (1.00-4.58)	1.19 (0.33-4.22)	0.784
Pharmacy	24 (11.21)	15 (7.94)	1.51 (0.69-3.33)	0.73 (0.20-2.56)	0.621
Radiologist	9 (4.21)	(63.17)	1.42 (0.46-4.39)	0.52 (0.08-3.19)	0.488
HMIS	15 (7.01)	11 (5.82)	1.29 (0.52-3.18)	0.48 (0.112.18)	0.349
Computer access					
Adequate	127 (59.35)	32 (16.93)	16.44 (8.89-30.39)	3.17 (1.19-8.46)	0.021^∗^
Inadequate	21 (9.81)	87 (46.03)	1.00		
HMIS training					
Yes	128 (68.45)	59 (31.22)	3.27 (2.17-4.94)	2.86 (1.22-6.71)	0.016^∗^
No	86 (39.81)	130 (68.78)	1.00		
EMR training					
Yes	172 (80.37)	43 (22.75)	13.90 (8.61-22.44)	4.00 (1.76-9.03)	0.001^∗∗^
No	42 (19.63)	146 (77.25)	1.00		
IT qualification					
Qualified	181 (84.58)	151 (45.48)	1.38 (0.82-2.30)	0.46 (0.19-1.10)	0.083
Not qualified	33 (15.42)	38 (20.11)	1.00		
Perceived system usability					
High usability	148 (80.87)	35 (19.13)	9.87 (6.18-15.75)	2.32 (0.87-3.56)	0.029
Low usability	66 (30.00)	154 (70.00)	1.00		
eHealth literacy					
High	195 (61.13)	124 (38.87)	5.37 (3.07-9.40)	0.94 (0.35-2.54)	0.916
Low	19 (22.62)	65 (77.38)	1.00		

Note: ^∗^significant at *p* value < 0.05 and ^∗∗^significant at *p* value < 0.01.

## Data Availability

Upon reasonable request from the corresponding author, the datasets created and/or analyzed during the current work will be available.

## Abbreviations

AOR: Adjusted Odds Ratio
COR: Crude Odds Ratio
D&M: DeLone and McLean
EMRs: Electronic Medical Records
ICT: Information and Communication Technology
IT: Information Technology.
